# Modeling the Impact of Climate Change on the Dynamics of Rift Valley Fever

**DOI:** 10.1155/2014/627586

**Published:** 2014-03-30

**Authors:** Saul C. Mpeshe, Livingstone S. Luboobi, Yaw Nkansah-Gyekye

**Affiliations:** ^1^School of CoCSE, Nelson Mandela African Institution of Science and Technology, P.O. Box 447, Arusha, Tanzania; ^2^Department of Mathematics, Makerere University, P.O. Box 7062, Kampala, Uganda

## Abstract

A deterministic SEIR model of rift valley fever (RVF) with climate change parameters was considered to compute the basic reproduction number *ℛ*_0_ and investigate the impact of temperature and precipitation on *ℛ*_0_. To study the effect of model parameters to *ℛ*_0_, sensitivity and elasticity analysis of *ℛ*_0_ were performed. When temperature and precipitation effects are not considered, *ℛ*_0_ is more sensitive to the expected number of infected *Aedes* spp. due to one infected livestock and more elastic to the expected number of infected livestock due to one infected *Aedes* spp. When climatic data are used, *ℛ*_0_ is found to be more sensitive and elastic to the expected number of infected eggs laid by *Aedes* spp. via transovarial transmission, followed by the expected number of infected livestock due to one infected *Aedes* spp. and the expected number of infected *Aedes* spp. due to one infected livestock for both regions Arusha and Dodoma. These results call for attention to parameters regarding incubation period, the adequate contact rate of *Aedes* spp. and livestock, the infective periods of livestock and *Aedes* spp., and the vertical transmission in *Aedes* species.

## 1. Introduction

Rift valley fever (RVF) is a viral disease that primarily affects animals (such as sheep, horses, cattle, goats, camels, and buffalos) and has the capacity to affect human beings. Rift valley fever virus (RVFV) is a member of the* Phlebovirus* genus family Bunyaviridae which has been isolated from at least 40 mosquito species in the filed and other arthropods [1, 2]. RVFV infection can cause severe disease in both animals and humans, leading to high disease induced death rate in livestock, long-term health effects in humans, and economic destruction of people [3, 4]. Currently, two types of vaccine for animals exist: a live vaccine and inactivated vaccine. However, the current live vaccine cannot be used for prevention, and prevention using the inactivated vaccine is difficult to sustain in RVF affected countries for economic reasons [3, 5].

RVF can be transmitted through an initial aerosol release and subsequent transmission through the mosquito vector. RVFV can remain dormant in* Aedes* spp. mosquito eggs in dry soil for years. During periods of heavy rainfall, larval habitats frequently become flooded, enabling the eggs to hatch and the mosquito population to rapidly increase, spreading the virus to animals on which they feed [6, 7]. Among animals, RVFV is spread primarily by the bite of infected mosquitoes, mainly* Aedes* and* Culex* spp. which can acquire the virus from feeding on an infected animal [8–10]. The female* Aedes* spp. mosquito is also capable of transmitting the virus directly to her offspring (vertical transmission) via eggs leading to new generations of infected mosquitoes hatching from eggs [11, 12]. This is not the case for* Culex* spp. mosquito.

RVFV can be transmitted to humans through the handling of animal tissue during slaughtering or butchering, assisting with animal births, conducting veterinary procedures, or from the disposal of carcasses or fetuses. Human infections have also resulted from the bites of infected mosquito vector, and by ingesting unpasteurized or uncooked milk and meat of infected animals [8–10, 13]. Transmission of RVFV by blood feeding flies is also possible. To date no human-to-human transmission of RVF has been documented [12].

RVF was first reported in Kenya (Africa) in 1931 [8], and it was primarily considered to be of sub-Saharan Africa until September, 2000, when RVF cases were confirmed in Saudi Arabia and Yemen (outside Africa) [14]. The recent outbreak in East Africa is that of 2006-2007 where 684 cases and 155 deaths were confirmed in Kenya, and 264 cases and 109 deaths in Tanzania. There were outbreaks also in Somalia and Sudan in the same period [12].

RVF outbreaks in East Africa have been largely correlated with the unusual heavy rainfall associated with El Ni$\ddot{\text{n}}$o [15], which consequently flooded many* Aedes* spp. breeding habitats. The hatching dynamics of* Aedes* spp. mosquitoes, the main reservoir of RVF in Africa, strongly depends on the rainfall pattern [1]. Eggs need to be flooded to hatch; thus, heavy rainfall results in a massive hatching episode and, consequently, the development of a large vector population. Once infection has been amplified in livestock, secondary vectors such as* Culex* spp. and other biting flies, which breed in semipermanent pools of water, become involved in the transmission of the virus [16].

Global temperature change, on the other hand, would affect the biology of the vectors, including feeding rate and egg production, and the length of the development cycle and the extrinsic incubation period. This may result in high vector density, an increased vector capacity to transmit the virus and a higher transmission rate [16]. When temperature rises above the biological maximum threshold for a species, it may decrease the vector population. Sustained climate shifts may lead to changes in the RVF burden in endemic areas and new outbreaks in areas of similar conditions. Thus, modeling the impact of climate change in the dynamics of RVF and its interventions is important for understanding of the disease.

Mathematical epidemiological models have been developed to assess the dynamics of RVF. Gaff et al. [17] proposed a theoretical model in a closed system which included two mosquito populations* Aedes* and* Culex* spp. and a livestock population. Their proposed model was a system of ordinary differential equations developed to explain the behaviour of the RVF transmission. The result of the development process was the production of a first-time model of this disease. The model was later modified by Gaff et al. [18] to assess the relative effectiveness of RVF countermeasures such as vector adulticide, vector larvicide, livestock vaccination, and livestock culling.

A theoretical model involving mosquito population, livestock and human population has been developed to study the dynamics of the disease using nonlinear differential equations [19]. The results show that the disease prevalence in both human and livestock is more sensitive to livestock and human recruitment rates suggesting isolation of livestock from human as a viable measure during the outbreak. The initial transmission and disease prevalence were found to be highly linked to mosquito population suggesting control measures such as vector adulticides and larvicides to be applied to reduce the mosquito population.

Fischer et al. [20] investigated the transmission potential of RVFV among livestock in the Netherlands. The model included the effect of temperature on the biting rate, mosquito population size, and the mortality of the vectors. The results show that high degree of vaccination and vector control strategy are needed to prevent RVF outbreaks. Other studies include that of Xue et al. [21] who developed a network-based metapopulation model approach to RVF epidemics to assess the disease spread in both time and space using network theory, Xue et al. [22] who investigate the spread of RVFV when introduced in United States, Chitnis et al. [23] who developed a model to assess the effect of vertical transmission in vector-borne disease with applications to RVF, and Niu et al. [24] who developed an epidemiological model of RVF with spatial dynamics to study the spatial effects.

In this paper, we propose a model that assesses the impact of climate change on the dynamics of RVF. The approach is based on the previous model of RVF transmission by Mpeshe et al. [19] and modifications have been made to incorporate vertical transmission and climate-driven parameters. To simplify the model, only temperature and precipitation are considered in this study. While* Aedes* spp. mosquito eggs are naturally infected by RVF virus via vertical transmission, this is not a case for* Culex* spp. mosquito and, therefore, we assume vertical transmission in our model only for* Aedes* species. To accommodate the impact of climate change we assert that temperature and precipitation can affect the laying and hatching of the eggs as well as the death rate, the effective contact rate, and the incubation period of the mosquitoes. When the epizootic is very high human can also be a source of infection for mosquitoes [25] and, therefore, also we assert in our model the human-to-mosquito transmission when the mosquitoes feed on an infected human.

## 2. Materials and Methods

### 2.1. Model Formulation

**Figure 1:**
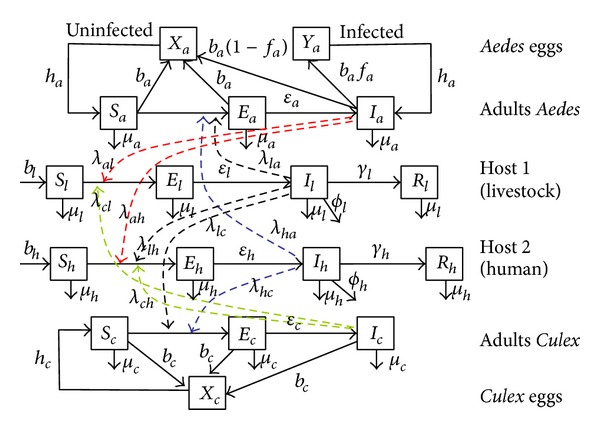
Flow diagram for the RVF model.

The model considers three populations: mosquitoes, livestock, and humans with disease-dependent death rate for livestock and humans. The mosquito population is subdivided into two:* Aedes* species and* Culex* species. Due to vertical transmission in* Aedes* spp., we include both infected and uninfected eggs in the model for determining the effect of vertical transmission in the initial transmission of RVF. The mode of transmission of RVF virus from vector to host, host to host, and host to vector is shown in the model flowchart shown by Figure 1. The egg population of* Aedes* spp. consists of uninfected eggs (*X*_*a*_) and infected eggs (*Y*_*a*_). The population for adult* Aedes* spp. consists of susceptible adults (*S*_*a*_), latently infected adults (*E*_*a*_), and infectious adults (*I*_*a*_). The egg population of* Culex* spp. consists of uninfected eggs (*X*_*c*_) only and the population for adult* Culex* spp. consists of susceptible adults (*S*_*c*_), latently infected adults (*E*_*c*_), and infectious adults (*I*_*c*_). The livestock population consists of susceptible livestock (*S*_*l*_), latently infected livestock (*E*_*l*_), infectious livestock (*I*_*l*_), and recovered livestock (*R*_*l*_). The human population consists of susceptible humans (*S*_*h*_), latently infected humans (*E*_*h*_), infectious humans (*I*_*h*_) and recovered humans (*R*_*h*_).  Table 1 shows the model parameters and their description as they have been used in this work. *T* and *P* represent temperature and precipitation, respectively.

The epidemiology cycle of RVF presented by Balenghien et al. [26] and Chevalier et al. [27] is here applied to develop the flow diagram shown by Figure 1. The inclusion of the transmission dynamics of RVF from* Aedes* spp. to human and vice versa is due to the fact that some* Aedes* spp. such as* Aedes vexans*,* Aedes aegpti*,* Aedes albopictus*,* Ae. ochraceus*,* Ae. mcintonshi*, and* Ae. dalzieli* and many others numerously feed on humans, and therefore has the capacity to cause infection to human [26–31].

Using the parameters in Table 1 and the model flow diagram in Figure 1, an SEIR model is derived on the basis of the explanations above using first-order nonlinear ordinary differential equations as follows:

*Aedes* Mosquito

(1a) $$\begin{array}{c} \frac{dX_{a}}{dt}=b_{a}\left(T,P\right)\left(N_{a}-f_{a}I_{a}\right)-h_{a}\left(T,P\right)X_{a}, \end{array}$$

(1b) $$\begin{array}{c} \frac{dY_{a}}{dt}=b_{a}\left(T,P\right)f_{a}I_{a}-h_{a}\left(T,P\right)Y_{a}, \end{array}$$

(1c) dSadt=ha(T,P)Xa−μa(T)Sa−λla(T)IlNlSa−λha(T)IhNhSa,

(1d) $$\begin{array}{c} \frac{dE_{a}}{dt}=\lambda_{la}\left(T\right)\frac{I_{l}}{N_{l}}S_{a}+\lambda_{ha}\left(T\right)\frac{I_{h}}{N_{h}}S_{a}-\left(\varepsilon_{a}\left(T\right)+\mu_{a}\left(T\right)\right)E_{a}, \end{array}$$

(1e) $$\begin{array}{c} \frac{dI_{a}}{dt}=h_{a}\left(T,P\right)Y_{a}+\varepsilon_{a}\left(T\right)E_{a}-\mu_{a}\left(T\right)I_{a}, \end{array}$$

(1f) $$\begin{array}{c} \frac{dN_{a}}{dt}=h_{a}\left(T,P\right)\left(X_{a}+Y_{a}\right)-\mu_{a}\left(T\right)N_{a}. \end{array}$$

*Culex* Mosquito

(2a) $$\begin{array}{c} \frac{dX_{c}}{dt}=b_{c}\left(T,P\right)N_{c}-h_{c}\left(T,P\right)X_{c}, \end{array}$$

(2b) dScdt=hc(T,P)Xc−μc(T)Sc−λlc(T)IlNlSc−λhc(T)IhNhSc,

(2c) $$\begin{array}{c} \frac{dE_{c}}{dt}=\lambda_{lc}\left(T\right)\frac{I_{l}}{N_{l}}S_{c}+\lambda_{hc}\left(T\right)\frac{I_{h}}{N_{h}}S_{c}-\left(\varepsilon_{c}\left(T\right)+\mu_{c}\left(T\right)\right)E_{c}, \end{array}$$

(2d) $$\begin{array}{c} \frac{dI_{c}}{dt}=\varepsilon_{c}\left(T\right)E_{c}-\mu_{c}\left(T\right)I_{c}, \end{array}$$

(2e) $$\begin{array}{c} \frac{dN_{c}}{dt}=h_{c}\left(T,P\right)X_{c}-\mu_{c}\left(T\right)N_{c}. \end{array}$$

Livestock

(3a) $$\begin{array}{c} \frac{dS_{l}}{dt}=b_{l}N_{l}-\mu_{l}S_{l}-\lambda_{al}\left(T\right)\frac{I_{a}}{N_{a}}S_{l}-\lambda_{cl}\left(T\right)\frac{I_{c}}{N_{c}}S_{l}, \end{array}$$

(3b) $$\begin{array}{c} \frac{dE_{l}}{dt}=\lambda_{al}\left(T\right)\frac{I_{a}}{N_{a}}S_{l}+\lambda_{cl}\left(T\right)\frac{I_{c}}{N_{c}}S_{l}-\left(\varepsilon_{l}+\mu_{l}\right)E_{l}, \end{array}$$

(3c) $$\begin{array}{c} \frac{dI_{l}}{dt}=\varepsilon_{l}E_{l}-\left(\mu_{l}+\phi_{l}+\gamma_{l}\right)I_{l}, \end{array}$$

(3d) $$\begin{array}{c} \frac{dR_{l}}{dt}=\gamma_{l}I_{l}-\mu_{l}R_{l}, \end{array}$$

(3e) $$\begin{array}{c} \frac{dN_{l}}{dt}=\left(b_{l}-\mu_{l}\right)N_{l}-\phi_{l}I_{l}. \end{array}$$

Humans

(4a) dShdt=bhNh−μhSh−λlhIlNlSh−λah(T)IaNaSh−λch(T)IcNcSh,

(4b) dEhdt=λlhIlNlSh+λah(T)IaNaSh+λch(T)IcNcSh−(εh+μh)Eh,

(4c) $$\begin{array}{c} \frac{dI_{h}}{dt}=\varepsilon_{h}E_{h}-\left(\mu_{h}+\phi_{h}+\gamma_{h}\right)I_{h}, \end{array}$$

(4d) $$\begin{array}{c} \frac{dR_{h}}{dt}=\gamma_{h}I_{h}-\mu_{h}R_{h}, \end{array}$$

(4e) $$\begin{array}{c} \frac{dN_{h}}{dt}=\left(b_{h}-\mu_{h}\right)N_{h}-\phi_{h}I_{h}. \end{array}$$

To test whether the model is well posed epidemiologically and mathematically, we need to investigate the feasibility of the model solution. Since *R*_*l*_ and *R*_*h*_ can be determined when *S*_*l*_, *S*_*h*_, *E*_*l*_, *E*_*h*_, *I*_*l*_, and *I*_*h*_ are known, without loss of generality, we omit the expression for *dR*_*l*_/*dt* and *dR*_*h*_/*dt* and write the system in compact form as

(5) $$\begin{array}{c} \frac{dX}{dt}=M\left(x\right)X+F, \end{array}$$

where *X* = (*X*_*a*_, *Y*_*a*_, *S*_*a*_, *E*_*a*_, *I*_*a*_, *X*_*c*_, *S*_*c*_, *E*_*c*_, *I*_*c*_, *S*_*l*_, *E*_*l*_, *I*_*l*_, *S*_*h*_, *E*_*h*_, *I*_*h*_)^*T*^,   *M*(*x*) is a 15 by 15 matrix, and *F* is a column matrix.

Substituting *I*_*a*_ = *N*_*a*_ − (*S*_*a*_ + *E*_*a*_) in *dX*_*a*_/*dt*, we have

(6) $$\begin{array}{c} \frac{dX_{a}}{dt}=b_{a}\left(T,P\right)N_{a}\left(1-f_{a}\right)+b_{a}f_{a}\left(S_{a}+E_{a}\right)-h_{a}\left(T,P\right)X_{a}, \end{array}$$

and, therefore,

(7) $$\begin{array}{c} M\left(x\right)=\left[\begin{array}{ccc} M_{1}\left(x\right) & 0 & 0\\ 0 & M_{2}\left(x\right) & 0\\ 0 & 0 & M_{3}\left(x\right) \end{array}\right], \end{array}$$

where

(8) M1(x)=[−ha(T,P)0ba(T,P)faba(T,P)fa00−ha(T,P)00ba(T,P)faha(T,P)0−(μa+A)0000A−(εa(T,P)+μa(T,P))00ha(T,P)0εa(T,P)−μa(T,P)],M2(x)=[−hc(T,P)000hc(T,P)−(μc(T,P)+B)000B−(εc(T,P)+μc(T,P))000εc(T,P)−μc(T,P)],M3(x)=[−(μl+C)00000C−(εl+μl)00000εl−(μl+ϕl+γl)000000−(μh+D)00000D−(εh+μh)00000εh−(μh+ϕh+γh)]

with

(9a) $$\begin{array}{c} A=\lambda_{la}\left(T\right)\frac{I_{l}}{N_{l}}+\lambda_{ha}\left(T\right)\frac{I_{h}}{N_{h}}, \end{array}$$

(9b) $$\begin{array}{c} B=\lambda_{lc}\left(T\right)\frac{I_{l}}{N_{l}}+\lambda_{hc}\left(T\right)\frac{I_{h}}{N_{h}}, \end{array}$$

(9c) $$\begin{array}{c} C=\lambda_{al}\left(T\right)\frac{I_{a}}{N_{a}}+\lambda_{cl}\left(T\right)\frac{I_{c}}{N_{c}}, \end{array}$$

(9d) $$\begin{array}{c} D=\lambda_{lh}\left(T\right)\frac{I_{l}}{N_{l}}+\lambda_{ah}\left(T\right)\frac{I_{a}}{N_{a}}+\lambda_{ch}\left(T\right)\frac{I_{c}}{N_{c}}, \end{array}$$

(10) F=(ba(T,P)Na(1−fa),0,0,0,0,bc(T,P)Nc, 0,0,0,blNl,0,0,bhNh,0,0)T≥0.

Combining all together, the matrix *M*(*x*) is a Metzler matrix for all ℝ_+_^15^. Therefore, the model system is positively invariant in ℝ_+_^15^, and *F* is Lipschitz continuous. Thus, the feasible region for the model system is the set

(11) 𝒟={(Xa,Ya,Sa,Ea,Ia,Xc,Sc,Ec,Ic,Sl,El,Il,Sh,Eh,Ih) ≥0∈ℝ+15}.

That is, the solution remains in the feasible region *𝒟* if it starts in this region. Hence, it is sufficient to study the dynamics of the model in *𝒟*.

### 2.2. Climate Driven Parameters

Several parameters related to mosquito vector, such as the hatching rate, vector mortality and longevity, biting rate, and extrinsic incubation period, depend on the temperature and precipitation. Using the existing studies and information from* Aedes vexans*,* Aedes aegypti*,* Culex pipiens*, and* Culex quinquefasciatus* [20, 32–34] which are potential vectors of RVF, we generalise the following relations for* Aedes* and* Culex* spp. mosquitoes.

#### 2.2.1. Hatching Rate or Mosquito Birth Rate, *h*(*T*,  *P*)

This is the number of eggs hatching into adult mosquitoes at a certain period of time which we also refer to as the mosquito birth rate. It will depend on the the daily survival probability *ρ* from eggs to adults and the duration *d* it takes to develop from eggs to adults. The daily survival probability is assumed to depend independently on temperature, precipitation/rainfall, and prolonged period of desiccation. Thus,

(12) $$\begin{array}{c} \rho \left(T,P,D\right)=\rho \left(T\right)\rho \left(P\right)\rho \left(D\right), \end{array}$$

where *ρ*(*T*) is the daily survival probability of immaturity due to temperature effect *T*; *ρ*(*P*) is the daily survival probability of immaturity due to precipitation effect *P*; and *ρ*(*D*) is the daily survival probability of immaturity due to desiccation effect *D*. The duration of maturation *d* is assumed to depend on temperature. Therefore, the hatching rate is given by

(13) $$\begin{array}{c} h\left(T,P,D\right)=\frac{\rho \left(T,P,D\right)}{d\left(T\right)}. \end{array}$$

#### 2.2.2. Survival Probability due to Temperature Effect *ρ*(*T*)

The daily survival probability *ρ*(*T*) is affected by the duration of maturation *d*(*T*) in exponential form, that is,

(14) $$\begin{array}{c} \rho \left(T\right)=\exp\left\{-\frac{1}{d\left(T\right)}\right\}. \end{array}$$

Fitting the data from [34] we obtain that 1/*d*(*T*) = *α*_1_ *T* ^2^ + *α*_2_ *T* + *α*_3_ for* Culex* spp. and 1/*d*(*T*) = *α*_1_ *T* ^3^ + *α*_2_ *T* ^2^ + *α*_3_ *T* + *α*_4_ for* Aedes* spp., where *α*_1_ = 0.0095, *α*_2_ = −0.4684, *α*_3_ = 5.8343 for* Culex* spp. and *α*_1_ = −0.0025, *α*_2_ = 0.2069, *α*_3_ = −5.5285, *α*_4_ = 48.2951 for* Aedes* spp.

#### 2.2.3. Survival Probability due to Precipitation Effect *ρ*(*P*)

Precipitation or rainfall is important in creating breeding sites for mosquitoes and causing massive hatching. But excessive rainfall increases mortality of immature due to flushing effect. Since rainfall has two effects, that is, positive and negative effect, we use the idea from [35] and assume the daily survival probability of immaturity due to precipitation effect to be

(15) $$\begin{array}{c} \rho \left(P\right)=\left(1-\exp\left\{-\beta_{1}\left(P-P_{1}\right)\right\}\right)\left(1-\exp\left\{-\beta_{2}\left(P_{2}-P\right)\right\}\right), \end{array}$$

where *β*_*i*_  (*i* = 1,2) are the sensitivity parameters; *P*_1_ is the minimum amount of rainfall to support maturity; and *P*_2_ is the maximum amount of rainfall which reduces their survival. For computational purposes we set *ρ*(*P*) = 0 for *P* < *P*_1_ and for *P* > *P*_2_.

#### 2.2.4. Survival Probability due to Desiccation Effect *ρ*(*D*)

Lack of precipitation affects the development of the immature. Following the approach by [32] we define the daily survival probability due desiccation as

(16) $$\begin{array}{c} \rho \left(D_{t}\right)=\frac{\exp\left(-\omega D_{t}\right)}{c+\exp\left(-\omega D_{t}\right)}, \end{array}$$

where *D* depends on precipitation *P* and is defined as

(17) $$\begin{array}{c} D_{t}=\{\begin{array}{cc} D_{t-1}+1 & P_{t}\leq P_{\text{th}}\\ 0 & \text{otherwise}, \end{array} \end{array}$$

where *P*_th_ is the threshold precipitation; *D*_*t*_ is the number of consecutive days up to time *t* when the precipitation *P*_*t*_ was below the threshold *P*_th_;   *ω* is the sensitivity parameter; and *c* is the constant that ensures that *ρ*(*D*_*t*_) is close to 1 at small values of *D*_*t*_.

#### 2.2.5. Daily Egg Laying Rate *b*(*T*)

The egg laying rate is assumed to depend on the moisture index. High moisture index correlates with high egg laying rate [33]. To model the daily egg laying rate we employ the equation derived by Gong et al. [33] that

(18) b(T,P)=Baseline  Egg  rate+Emax⁡1+exp⁡⁡{−(Moisture  Index−Emean)/Evar},

where Baseline Egg rate is the baseline for fecundity, *E*_max⁡_ is the maximum daily egg laying rate, *E*_mean_ is the mean of daily egg laying rate, and *E*_var_ is the variance function.

To compute the moisture index, we apply Thornthwaite's moisture index [36] that

(19) $$\begin{array}{c} \text{Moisture}\;\text{Index}\left(I_{m}\right)=100\left(\frac{r}{E_{0}}-1\right), \end{array}$$

where *r* is the precipitation rate, and *E*_0_ is the potential evapotranspiration. In absence of the potential evapotranspiration, Linacre's method [37] can be applied. That is,

(20) $$\begin{array}{c} E_{0}=\frac{700T_{m}/\left(100-A\right)+15\left(T-T_{d}\right)}{\left(80-T\right)}\text{m}{\text{mday}}^{-1}, \end{array}$$

where, *T*_*m*_ = *T* + 0.00*h* with *h* being the elevation (metres), *T* is the mean temperature, *A* is the latitude (degrees), and *T*_*d*_ is the mean dew-point.

#### 2.2.6. Longevity of Mosquitoes 1/*μ*(*T*)

Different studies show that the longevity of mature mosquitoes also depends on the temperature. To model the longevity, equations deduced by Fischer et al. [20] are applied. That is,

(21) $$\begin{array}{c} \frac{1}{\mu \left(T\right)}=a_{0}-a_{1}T, \end{array}$$

where *a*_0_ = 25.8, *a*_1_ = 0.45 for* Aedes* spp., and *a*_0_ = 69.1, *a*_1_ = 2.14 for* Culex* spp.

#### 2.2.7. Extrinsic Incubation Period of Mosquitoes 1/*ɛ*(*T*)

Extrinsic incubation period is the time between a blood meal on an infections host and the first successful transmission from vector to host during another blood meal. We also adapt the expressions by Fischer et al. [20]. That is,

(22) $$\begin{array}{c} \frac{1}{ɛ\left(T\right)}=\varepsilon_{\max}-\varepsilon_{\text{slope}}T, \end{array}$$

where *ε*_max⁡_ = 18.9, *ε*_slope_ = 0.30 for* Aedes* spp., and *ε*_max⁡_ = 11.3,   *ε*_slope_ = 0.30 for* Culex* spp.

#### 2.2.8. Adequate Contact Rate *λ*(*T*)

Adequate contact rate is contact which is sufficient for transmission of the infection from an infective to a susceptible. Thus, in this study

(23) adequate  contact  rate  =biting  rate×  probability  of  transmission.

The biting rate depends on temperature, and we assume a linear relationship as in Fischer et al. [20]. That is,

(24) $$\begin{array}{c} a\left(T\right)=a_{\text{slope}}\left(T-T_{\min}\right), \end{array}$$

where *a*_slope_ = 0.0173, *T*_min⁡_ = 9.60 for all mosquito species. Assume that the probability of transmission is independent to temperature, we have

(25a) $$\begin{array}{c} \lambda_{al}\left(T\right)=a_{\text{slope}}\left(T-T_{\min}\right)\rho_{al},\;\;\rho_{al}=0.70, \end{array}$$

(25b) $$\begin{array}{c} \lambda_{cl}\left(T\right)=a_{\text{slope}}\left(T-T_{\min}\right)\rho_{cl},\;\;\rho_{cl}=0.78, \end{array}$$

(25c) $$\begin{array}{c} \lambda_{la}\left(T\right)=a_{\text{slope}}\left(T-T_{\min}\right)\rho_{la},\;\;\rho_{la}=0.38, \end{array}$$

(25d) $$\begin{array}{c} \lambda_{lc}\left(T\right)=a_{\text{slope}}\left(T-T_{\min}\right)\rho_{lc},\;\rho_{lc}=0.22, \end{array}$$

(25e) $$\begin{array}{c} \lambda_{ah}\left(T\right)=a_{\text{slope}}\left(T-T_{\min}\right)\rho_{ah},\;\rho_{ah}=0.01, \end{array}$$

(25f) $$\begin{array}{c} \lambda_{ha}\left(T\right)=a_{\text{slope}}\left(T-T_{\min}\right)\rho_{ha},\;\;\rho_{ha}=0.05, \end{array}$$

(25g) $$\begin{array}{c} \lambda_{ch}\left(T\right)=a_{\text{slope}}\left(T-T_{\min}\right)\rho_{ch},\;\rho_{ch}=0.01, \end{array}$$

(25h) $$\begin{array}{c} \lambda_{hc}\left(T\right)=a_{\text{slope}}\left(T-T_{\min}\right)\rho_{hc},\;\rho_{hc}=0.015. \end{array}$$

### 2.3. The Basic Reproduction Number

The basic reproduction number *ℛ*_0_ is computed using the method of next generation matrix as outlined by [38]. Let *k*_*ij*_ be the expected number of the new cases of type *i* caused by one infected individual of type *j*, during the entire period of infectiousness. Define a matrix *K* whose entries are *k*_*ij*_, that is, *K* = [*k*_*ij*_]. Then, *ℛ*_0_ = *ρ*(*K*), where *ρ*(*K*) is spectral radius of *K*. For our model, we define four type-at-infection consisting of two vectors and two hosts, namely,* Aedes* spp. (type 1),* Culex* spp. (type 2), livestock (type 3), and humans (type 4). The resulting next generation matrix is

(26) $$\begin{array}{c} K=\left[\begin{array}{cccc} k_{11} & k_{12} & k_{13} & k_{14}\\ k_{21} & k_{22} & k_{23} & k_{24}\\ k_{31} & k_{32} & k_{33} & k_{34}\\ k_{41} & k_{42} & k_{43} & k_{44} \end{array}\right], \end{array}$$

where *k*_11_ is the expected number of infected eggs laid by* Aedes* spp. via transovarial transmission, *k*_12_ is the expected number of infected* Aedes* spp. due to one infected* Culex*, *k*_21_ is the expected number of infected* Culex* spp. due to one infected* Aedes* spp., *k*_13_ is the expected number of infected* Aedes* spp. due to one infected livestock, *k*_31_ is the expected number of infected livestock due to one infected* Aedes* spp., *k*_14_ is the expected number of infected* Aedes* spp. due to one infected human, *k*_41_ is the expected number of infected humans due to one infected* Aedes* spp., *k*_22_ is the expected number of infected eggs laid by* Culex* spp. via transovarial transmission, *k*_23_ is the expected number of infected* Culex* spp. due to one infected livestock, *k*_32_ is the expected number of infected livestock due to one infected* Culex* spp., *k*_24_ is the expected number of infected* Culex* spp. due to one infected human, *k*_42_ is the expected number of infected humans due to one infected* Culex* spp., *k*_33_ is the expected number of infected livestock due to one infected livestock, *k*_34_ is the expected number of infected livestock due to one infected human, *k*_43_ is the expected number of infected humans due to one infected livestock, and and *k*_44_ is the expected number of infected humans due to one infected human.

Since there is no vertical transmission in* Culex* spp., then *k*_22_ = 0. The same applies for *k*_33_ and *k*_44_. Also* Aedes* spp. cannot infect* Culex* spp. and vice versa; therefore, *k*_12_ = *k*_21_ = 0. Humans cannot infect livestock, so *k*_34_ = 0. Hence, we have

(27) $$\begin{array}{c} K=\left[\begin{array}{cccc} k_{11} & 0 & k_{13} & k_{14}\\ 0 & 0 & k_{23} & k_{24}\\ k_{31} & k_{32} & 0 & 0\\ k_{41} & k_{42} & k_{43} & 0 \end{array}\right]. \end{array}$$

The entry *k*_*ij*_ depends on the probability that the individual of type *j* survives the incubation, the adequate contact rate: individual type *j* to individual type *i*, and the infective period of individual of type *j*. For example, *k*_13_ will depend on the probability that livestock survives the incubation period, the adequate contact rate from livestock to* Aedes* spp., and the infective period of livestock. We therefore derive the *k*_*ij*_ values as follows:

(28a)k11=ba(T,P)faμa,  k13=(εlεl+μl)(λla(T)μl+ϕl+γl),(28b)k14=(εhεh+μh)(λha(T)μh+ϕh+γh),k23=(εlεl+μl)(λlc(T)μl+ϕl+γl),(28c)k24=(εhεh+μh)(λhc(T)μh+ϕh+γh),k31=(εa(T)εa(T)+μa(T))(λal(T)μa(T)),(28d)k32=(εc(T)εc(T)+μc(T))(λcl(T)μc(T)),k41=(εa(T)εa(T)+μa(T))(λah(T)μa(T)),(28e)k42=(εc(T)εc(T)+μc(T))(λch(T)μc(T)),k43=(εlεl+μl)(λlh(T)μl+ϕl+γl).

### 2.4. Sensitivity and Elasticity Analyses of *ℛ*_0_

Sensitivities quantify how *ℛ*_0_ changes in response to the small shifts in the value of a parameter, while elasticities quantify the proportional change in *ℛ*_0_ in response to the proportional change in a parameter. Both sensitivity and elasticity values can be used to judge which parameters are important to measure accurately and where variation in parameters will translate into variation in *ℛ*_0_.

Caswell [39] developed a way to quantify sensitivity and elasticity of the growth rate *λ* to changes in vital rates *a*_*ij*_ where *a*_*ij*_ are the entries of population matrix *A*. That is, the sensitivity of the growth rate *λ* to changes in vital rates *a*_*ij*_ is given by

(29) $$\begin{array}{c} s_{ij}=\frac{\partial \lambda }{\partial a_{ij}}=\frac{v_{i}w_{j}}{\langle w,v\rangle }, \end{array}$$

where** w** and** v** are the right and left eigenvectors, respectively, corresponding to the dominant eigenvalue *λ* of the matrix *A*, and 〈**w**, **v**〉 is the dot product of** w** and** v**. In case *a*_*ij*_ is a function of other lower-level parameters, then, the chain rule can be applied to estimate the sensitivity of *λ* to changes in any model parameter *p* as

(30) $$\begin{array}{c} s\left(p\right)=\frac{\partial \lambda }{\partial p}=\sum_{ij}\frac{\partial \lambda }{\partial a_{ij}}\frac{\partial a_{ij}}{\partial p}. \end{array}$$

The elasticity of the growth rate *λ* to changes in vital rates *a*_*ij*_, the entries of population matrix *A*, is given by

(31) $$\begin{array}{c} e_{ij}=\frac{\partial \log\lambda }{\partial \log a_{ij}}=\frac{a_{ij}}{\lambda }\frac{\partial \lambda }{\partial a_{ij}}. \end{array}$$

For *a*_*ij*_ a function of other lower-level parameters *p*, the elasticity is given by

(32) $$\begin{array}{c} e\left(p\right)=\frac{p}{\lambda }\frac{\partial \lambda }{\partial p}=\frac{p}{\lambda }\sum_{ij}\frac{\partial \lambda }{\partial a_{ij}}\frac{\partial a_{ij}}{\partial p}. \end{array}$$

The theory of sensitivity analysis developed for the matrix models by Caswell [39] can be extended to the disease models to study the sensitivity and elasticity of *ℛ*_0_ to the changes in the reproduction numbers *k*_*ij*_ or the parameters defining them. Thus, the sensitivity *s*_*ij*_ of a matrix element *k*_*ij*_ is defined as the change in the eigenvalue (*ℛ*_0_) due to change in *k*_*ij*_ given by

(33) $$\begin{array}{c} s_{ij}=\frac{\partial {ℛ}_{0}}{\partial k_{ij}}. \end{array}$$

For individual parameter, the sensitivity *s*(*p*) is given by

(34) $$\begin{array}{c} s\left(p\right)=\sum_{ij}\frac{\partial {ℛ}_{0}}{\partial k_{ij}}\frac{\partial k_{ij}}{\partial p}. \end{array}$$

The elasticity *e*_*ij*_ of a matrix element *k*_*ij*_ is defined as

(35) $$\begin{array}{c} e_{ij}=\frac{k_{ij}}{{ℛ}_{0}}\frac{\partial {ℛ}_{0}}{\partial k_{ij}}. \end{array}$$

For individual parameters *p*, the elasticity is given by

(36) $$\begin{array}{c} e\left(p\right)=\frac{p}{{ℛ}_{0}}\sum_{ij}\frac{\partial {ℛ}_{0}}{\partial k_{ij}}\frac{\partial k_{ij}}{\partial p}. \end{array}$$

In order to study the impact of climate change to climate-driven parameter in the distribution of *ℛ*_0_ we use climate data from two different regions in Tanzania, namely, Arusha and Dodoma for the 2006-2007 outbreak. According to WHO [12], RVF was reported in 10 out of the 21 regions of Tanzania where 12 cases were reported in Arusha region, 1 in Dar es Salaam, 156 in Dodoma, 4 in Iringa, 6 in Manyara, 50 in Morogoro, 5 in Mwanza, 5 in the Pwani, 24 in Singida, and 1 in Tanga regions. From the data we find that Dodoma has more than 50% of the total cases giving a justification for being a case of study, and Arusha is considered in this study because the first case was reported in January 2007 in this region.

## 3. Results and Discussion

In this section we first present the result for *ℛ*_0_ when the parameters are assumed to be independent of climate change. Then, we will compute the numerical value for *ℛ*_0_ when climate change is considered to climate-driven parameters. Sensitivity and elasticity analysis results in both cases will be presented.  Table 2 shows the parameter values for low range and high range which are used to compute the numerical value for *ℛ*_0_ when temperature and precipitation effects are not considered.

When we substitute the values in Table 2 to the expressions of the elements of matrix *K* and compute *ℛ*_0_, we obtain that for low parameter values *ℛ*_0_ = 0.1941 and for high parameter values *ℛ*_0_ = 6.8071.

**Figure 2:**
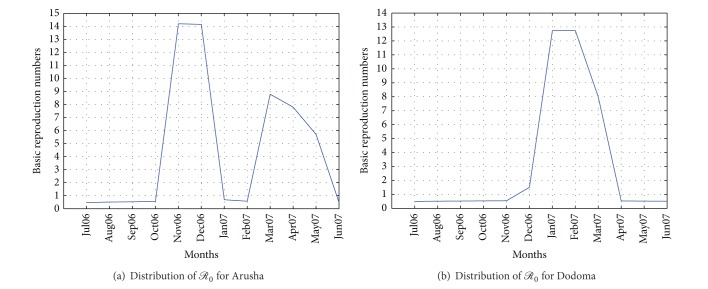
Distribution of *ℛ*_0_ for climatic data in Arusha and Dodoma.

When climate change parameters were evaluated using the climate variable the value of *ℛ*_0_ change from 0.4747 to 14.2007 in Arusha with the highest value marked in November 2006 ( = 14.2007) followed by December 2006 ( = 14.1530). The value of *ℛ*_0_ dropped below 1 in January 2007 and February 2007, but it rose again in March, April, and May.  Figure 2(a) shows the distribution of *ℛ*_0_ from July 2006 to June 2007 in Arusha region.

In Dodoma, the highest *ℛ*_0_ was marked February 2007 ( = 12.7438) followed by January 2007 ( = 12.7368) then March 2007 ( = 7.9899) and December 2006 ( = 1.5088) as Figure 2(b) indicates.

**Figure 3:**
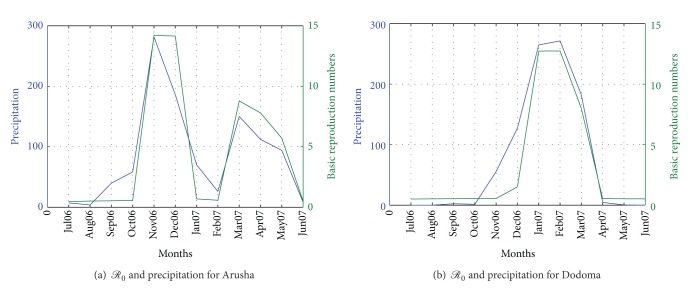
*ℛ*_0_ and precipitation for climatic data in Arusha and Dodoma.

**Figure 4:**
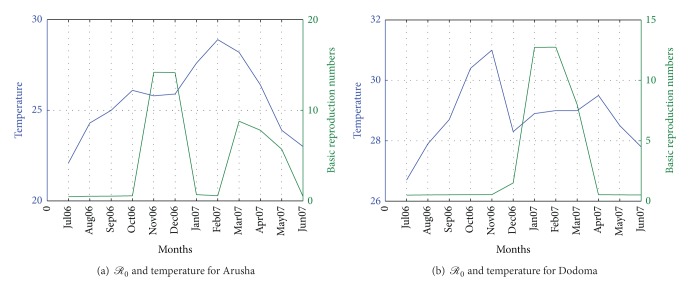
*ℛ*_0_ temperature for climatic data in Arusha and Dodoma.

While it is clear that *ℛ*_0_ increases with increase in rainfall, it is not the case for temperature where we experience high *ℛ*_0_ for low temperatures.  Figure 3 shows the plots for *ℛ*_0_ and precipitation over months, and Figure 4 shows the plots for *ℛ*_0_ and temperature over months.

**Figure 5:**
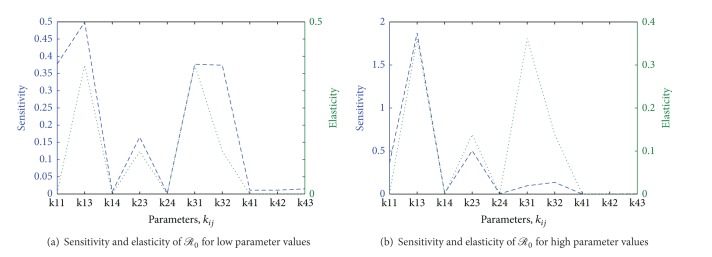
Sensitivity and elasticity of *ℛ*_0_ plotted against the low and high parameters values.

Table 3 shows the sensitivity and elasticity values of *ℛ*_0_, to both low and high parameter values. For both low and high parameter values, *ℛ*_0_ is most sensitive to *k*_13_, the expected number of infected* Aedes* spp. Due to one infected livestock, and to and most elastic to *k*_31_, the expected number of infected livestock due to one infected* Aedes* spp. Table 3 shows the sensitivity and elasticity values of *ℛ*_0_ for low and high parameter values, and Figure 5 shows the plots of sensitivity and elasticity values plotted against the parameter *k*_*ij*_. The results suggest that attention should be given to parameters regarding incubation period, the adequate contact rate, and the infective period of livestock and* Aedes* spp.

**Figure 6:**
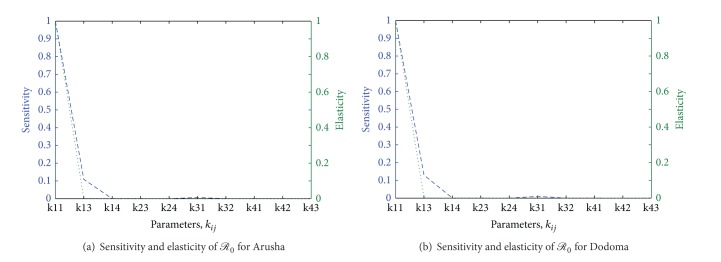
Sensitivity and elasticity of *ℛ*_0_ plotted against the parameters *k*_*ij*_ for climatic data in Arusha and Dodoma.

When climatic data are used, *ℛ*_0_ is found to be more sensitive and elastic to *k*_11_, the expected number of infected eggs laid by* Aedes* spp. via transovarial transmission, followed by *k*_13_ and *k*_31_ for both regions Arusha and Dodoma.  Table 4 shows the sensitivity and elasticity values of *ℛ*_0_ for Dodoma and Arusha climate data, and Figure 6 shows the plots of sensitivity and elasticity against the parameters *k*_*ij*_. The results call for attention to parameters regarding incubation period, the adequate contact rate of* Aedes* spp. and livestock, the infective periods of livestock and* Aedes* spp., and the vertical transmission in* Aedes* spp.

## 4. Conclusion

A deterministic SEIR model of RVF has been presented to study the impact of climate change variables mainly temperature and precipitation. The model presented here is just a simple representation of the complex ecological situation involved in the epidemiology of RVF. The formulation of the model, computation of *ℛ*_0_, and sensitivity and elasticity analyses of *ℛ*_0_ are based on the assumptions made to build the model as well as the chosen parameter values. Real climate data from Dodoma and Arusha where outbreak occured in 2006-2007 have been used to study the distribution of *ℛ*_0_ in the whole period of the outbreak. Though the current analysis presented in this work may not be exhaustible, it remains, however, an important step toward the study of the impact of climate change on the dynamics of RVF.

## Figures and Tables

**Table 1:** Parameters used in the model formulation and their description.

Parameter	Description	Dependent on climate change
1/*h*_*a*_(*T*, *P*)	Development time of *Aedes* mosquitoes	Temperature and precipitation
1/*h*_*c*_(*T*, *P*)	Development rate of *Culex* mosquitoes	Temperature and precipitation
*b*_*a*_(*T*, *P*)	Number of *Aedes* eggs laid per day	Temperature and precipitation
*b*_*c*_(*T*, *P*)	Number of *Culex* eggs laid per day	Temperature and precipitation
*b*_*h*_	Daily birth rate in humans	Not considered
*b*_*l*_	Daily birth rate in livestock	Not considered
1/*μ*_*a*_(*T*)	Lifespan of *Aedes* mosquitoes	Temperature
1/*μ*_*c*_(*T*)	Lifespan of *Culex* mosquitoes	Temperature
1/*μ*_*h*_	Lifespan of humans	Not considered
1/*μ*_*l*_	Lifespan of livestock	Not considered
1/ε_*a*_(*T*)	Incubation period of *Aedes* mosquitoes	Temperature
1/ε_*c*_(*T*)	Incubation period of *Culex* mosquitoes	Temperature
1/ε_*h*_	Incubation period of humans	Not considered
1/ε_*l*_	Incubation period of livestock	Not considered
ϕ_*l*_	Death rate of livestock due to disease	Not considered
ϕ_*h*_	Death rate of humans due to disease	Not considered
1/γ_*l*_	Infectious period in livestock	Not considered
1/γ_*h*_	Infectious period in humans	Not considered
λ_al_(*T*)	Adequate contact rate: *Aedes* to livestock	Temperature
λ_cl⁡_(*T*)	Adequate contact rate: *Culex* to livestock	Temperature
λ_la_(*T*)	Adequate contact rate: livestock to *Aedes *	Temperature
λ_lc_(*T*)	Adequate contact rate: livestock to *Culex *	Temperature
λ_ah_(*T*)	Adequate contact rate: *Aedes* to humans	Temperature
λ_ch_(*T*)	Adequate contact rate: *Culex* to humans	Temperature
λ_ha_(*T*)	Adequate contact rate: humans to *Aedes *	Temperature
λ_hc_(*T*)	Adequate contact rate: humans to *Culex *	Temperature
λ_lh_	Adequate contact rate: livestock to humans	Not considered
*f*_*a*_	Vertical transmission rate in *Aedes *	Not considered

**Table 2:** Parameters with their estimated lower and higher values without considering impact of climate change.

Parameter	low value	high value	Reference
1/*b*_*a*_	100	200	Assumed
1/*μ*_*a*_	3 days	60 days	[17]
1/*μ*_*c*_	3 days	60 days	[17]
1/*μ*_*h*_	40 yrs	60 yrs	[19]
1/*μ*_*l*_	1 yr	10 yrs	[40]
1/ε_*a*_	4 days	8 days	[19]
1/ε_*c*_	4 days	8 days	[19]
1/ε_*h*_	2 day	6 days	[19]
1/ε_*l*_	1 day	6 days	[19]
ϕ_*l*_	0.025	0.10	[19]
ϕ_*h*_	0.01	0.10	[19]
*f*_*a*_	0.05	0.1	[18]
1/γ_*l*_	1 day	5 days	[19]
1/γ_*h*_	4 days	7 days	[19]
λ_*al*_	0.15	0.48	[18]
λ_cl⁡_	0.05	0.13	[18]
λ_*la*_	0.15	0.395	[18]
λ_*lc*_	0.15	0.56	[18]
λ_*ah*_	0.001	0.002	[22]
λ_*ch*_	0.0005	0.001	[22]
λ_*ha*_	0.001	0.0015	Assumed
λ_*hc*_	0.0015	0.002	Assumed
λ_*lh*_	0.001	0.002	[19]

**Table 3:** Sensitivity and elasticity of *ℛ*_0_ for low and high parameter values.

Parameter	Sensitivity	Elasticity
Low parameter values		
*k*_11_	0.37750	0.00292
*k*_13_	0.49913	0.37438
*k*_14_	0.00629	0.00012
*k*_23_	0.16513	0.12386
*k*_24_	0.00208	0.00006
*k*_31_	0.37679	0.37443
*k*_32_	0.37399	0.12388
*k*_41_	0.01118	0.00007
*k*_42_	0.01109	0.00003
*k*_43_	0.01497	0.00008

High parameter values		
*k*_11_	0.36274	0.00160
*k*_13_	1.87148	0.36107
*k*_14_	0.01149	0.00001
*k*_23_	0.50469	0.13804
*k*_24_	0.00310	0.00000
*k*_31_	0.09672	0.36107
*k*_32_	0.13653	0.13804
*k*_41_	0.00045	0.00001
*k*_42_	0.00063	0.00000
*k*_43_	0.00231	0.00000

**Table 4:** Sensitivity and elasticity of *ℛ*_0_ for Dodoma and Arusha climate data.

Parameter	Sensitivity	Elasticity
Dodoma		
*k*_11_	0.99874	0.99748
*k*_13_	0.12996	0.00126
*k*_14_	0.00020	0.00000
*k*_23_	0.00010	0.00000
*k*_24_	0.00000	0.00000
*k*_31_	0.00971	0.00126
*k*_32_	0.00001	0.00000
*k*_41_	0.00010	0.00000
*k*_42_	0.00000	0.00000
*k*_43_	0.00001	0.00000

Arusha		
*k*_11_	0.99921	0.99841
*k*_13_	0.10903	0.00079
*k*_14_	0.00016	0.00000
*k*_23_	0.00012	0.00000
*k*_24_	0.00000	0.00000
*k*_31_	0.00727	0.00079
*k*_32_	0.00000	0.00000
*k*_41_	0.00008	0.00000
*k*_42_	0.00000	0.00000
*k*_43_	0.00001	0.00000

## References

[1] Meegan JM, Bailey CL, Monath TH (1988). Rift Valley fever. *The Arboviruses: Epidemiology and Ecology*.

[2] Moutailler S, Krida G, Schaffner F, Vazeille M, Failloux A-B (2008). Potential vectors of rift valley fever virus in the Mediterranean region. *Vector-Borne and Zoonotic Diseases*.

[3] FAO Rift Valley Fever: Vigilance Needed in the Coming Months.

[4] Peters CJ, Linthicum KJ, Beran GB (1994). Rift Valley fever. *Handbook of Zoonoses*.

[5] Pepin M, Bouloy M, Bird BH, Kemp A, Paweska J (2010). Rift Valley fever virus (Bunyaviridae: Phlebovirus): An update on pathogenesis, molecular epidemiology, vectors, diagnostics and prevention. *Veterinary Research*.

[6] Linthicum KJ, Davies FG, Bailey CL, Kairo A (1983). A mosquito species succession Dambo in an East African forest. *Mosquito News*.

[7] Linthicum KJ, Davies FG, Bailey CL, Kairo A (1983). Mosquito species encountered in a flooded grassland Dambo in Kenya. *Mosquito News*.

[8] Daubney R, Hudson JR, Garnham PC (1931). Enzootic hepatitis of Rift Valley fever, an undescribed virus disease of sheep, cattle and man from East Africa. *Journal of Pathology and Bacteriology*.

[9] Davies FG (1975). Observations on the epidemiology of Rift Valley fever in Kenya. *Journal of Hygiene*.

[10] Scott GR, Weddell W, Reid D (1956). Preliminary finding on the prevalence of Rift Valley fever in Kenya Cattle. *Bulletin of Epizootic Diseases of Africa*.

[11] Favier C, Chalvet-Monfray K, Sabatier P, Lancelot R, Fontenille D, Dubois MA (2006). Rift Valley fever in West Africa: the role of space in endemicity. *Tropical Medicine and International Health*.

[12] WHO (2007). Rift Valley fever. *Fact Sheet*.

[13] Davies FG, Martin V (2006). Recognising Rift Valley fever. *Veterinaria Italiana*.

[14] Jupp PG, Kemp A, Grobbelaar A (2002). The 2000 epidemic of Rift Valley fever in Saudi Arabia: Mosquito vector studies. *Medical and Veterinary Entomology*.

[15] Linthicum KJ, Anyamba A, Tucker CJ, Kelley PW, Myers MF, Peters CJ (1999). Climate and satellite indicators to forecast Rift Valley fever epidemics in Kenya. *Science*.

[16] Martini V, Chevalier V, Ceccato P (2008). The impact of climate change on the epidemiology and control of Rift Valley fever. *Revue Scientifique et Technique*.

[17] Gaff HD, Hartley DM, Leahy NP (2007). An epidemiological model of rift valley fever. *Electronic Journal of Differential Equations*.

[18] Gaff H, Burgese C, Jackson J, Niu T, Papelis Y, Hartley D (2011). Mathematical model to assess the relative effectiveness of Rift Valley fever countermeasures. *International Journal of Artificial Life Research*.

[19] Mpeshe SC, Haario H, Tchuenche JM (2011). A mathematical model of Rift Valley fever with human host. *Acta Biotheoretica*.

[20] Fischer EAJ, Boender GJ, de Koeijer AA, Nodelijk HA, van Roermund HJ (2013). The transmission potential of Rift Valley fever virus among livestock in the Netherlands: a modelling study. *Veterinary Research*.

[21] Xue L, Scott HH, Scoglio C (2012). A Network based Meta population approach to model Rift Valley epidemics. *Journal of Theoretical Biology*.

[22] Xue L, Scott HH, Cohnstaedt LW, Scoglio C (2013). A hierarchical network approach for modeling Rift Valley fever epidemics. *PLoS ONE*.

[23] Chitnis N, Hyman JM, Manore CA (2013). Modeling vertical transmision in vector-borne disease with applications to Rift Valley Fever. *Journal of Biological Dynamics*.

[24] Niu N, Gaff HD, Papelis YE, Hartley DM (2012). An epidemiological model of Rift Valley fever with spatial dynamics. *Computational and Mathematical Methods in Medicine*.

[25] Spickler AR Rift Valley Fever: infectious enzootic hepatitis of sheep and cattle. http://www.cfsph.iastate.edu/Factsheets/pdfs/rift_valley_fever.pdf.

[26] Balenghien T, Cardinale E, Chevalier V (2013). Towards a better understanding of rift valley fever epidemiology in the South-West of the Indian Ocean. *Vetenary Research*.

[27] Chevalier V, Pépin M, Plée L, Lancelot R (2010). Rift Valley fever: a threat for Europe?. *Euro Surveillance*.

[28] Edman JD, Takken W, Scott TW (2003). Fitness advantages in multiple blood-feeding: the *Aedes aegypti* example. *Ecological Aspects For Application of Genetically Modified Mosquitoes*.

[29] Fontenille D, Traore-Lamizana M, Diallo M, Thonnon J, Digoutte JP, Zeller HG (1998). New vectors of Rift Valley fever in West Africa. *Emerging Infectious Diseases*.

[30] Kamgang B, Nchoutpouen E, Simard F, Paupy C (2012). Notes on the blood-feeding behavior of *Aedes albopictus* (Diptera: Culicidae) in Cameroon. *Parasites and Vectors*.

[31] Le Coupaner A, Babin D, Fiette L (2013). *Aedes* mosquito saliva modulates Rift Valley fever pathogenicity. *PLoS Neglected Tropical Diseases*.

[32] Ahumada JA, Lapointe D, Samuel MD (2004). Modeling the population dynamics of *Culex quinquefasciatus* (Diptera: Culicidae), along an elevational gradient in Hawaii. *Journal of Medical Entomology*.

[33] Gong H, De Gaetano A, Harrington L A climate based mosquito population model.

[34] Rueda LM, Patel KJ, Axtell RC, Stinner RE (1990). Temperature-dependent development and survival rates of *Culex quinquefasciatus* and *Aedes aegypti* (Diptera: Culicidae). *Journal of Medical Entomology*.

[35] Shi P, Ge F, Sun Y, Chen C (2011). A simple model for describing the effect of temperature on insect developmental rate. *Journal of Asia-Pacific Entemology*.

[36] Thornthwaite C, Mather J (1955). The water balance. *Meteorology*.

[37] Linacre ET (1977). A simple formula for estimating evaporation rates in various climates, using temperature data alone. *Agricultural Meteorology*.

[38] Castillo-Chavez C, Feng Z, Huang W, Castillo-Chavez C, van den Driessche P, Kirschner D, Yakubu AA (2002). On the computation of *ℛ*
_0_ and its role in global stability. *Mathematical Approaches for Emerging and reemerging Infection Diseases: An Introduction*.

[39] Caswell H (2001). *Matrix Population Models: Construction, Analysis, and Interpretation*.

[40] Radostits O (2001). *Herd Healthy: Food Animal Production Medicine*.

